# The Effects of Hyperacute Serum on the Elements of the Human Subchondral Bone Marrow Niche

**DOI:** 10.1155/2018/4854619

**Published:** 2018-04-16

**Authors:** Melinda Simon, Bálint Major, Gabriella Vácz, Olga Kuten, István Hornyák, Adél Hinsenkamp, Dorottya Kardos, Marcell Bagó, Domonkos Cseh, Adrienn Sárközi, Denes Horvathy, Stefan Nehrer, Zsombor Lacza

**Affiliations:** ^1^Institute of Clinical Experimental Research, Semmelweis University, Budapest, Hungary; ^2^Department of Orthopedics, Polyclinic of the Hospitaller Brothers of St. John of God in Budapest, Budapest, Hungary; ^3^Orthosera GmbH, Krems an der Donau, Austria; ^4^Danube University Krems, Krems an der Donau, Austria; ^5^Department of Physiology, Semmelweis University, Budapest, Hungary

## Abstract

Mesenchymal stem cells (MSCs) are widely used in laboratory experiments as well as in human cell therapy. Their culture requires animal sera like fetal calf serum (FCS) as essential supplementation; however, animal sera pose a risk for clinical applications. Human blood derivatives, for example, platelet-rich plasma (PRP) releasates, are potential replacements of FCS; however, it is unclear which serum variant has the best effect on the given cell or tissue type. Additionally, blood derivatives are commonly used in musculoskeletal diseases like osteoarthritis (OA) or osteonecrosis as “proliferative agents” for the topical MSC pool. Hyperacute serum (HAS), a new serum derivative, has been designed to approximate the natural coagulation cascade with a single-step, additive-free preparation method. We investigated the effects of HAS on monolayer MSC cultures and in their natural niche, in 3D subchondral bone and marrow explants. Viability measurements, RT-qPCR evaluation for gene expression and flow cytometry for cell surface marker analysis were performed to compare the effects of FCS-, PRP-, or HAS-supplemented culture media. Monolayer MSCs showed significantly higher metabolic activity following 5 days' incubation in HAS, and osteoblast-specific mRNA expression was markedly increased, while cells also retained their MSC-specific cell surface markers. A similar effect was observed on bone and marrow explants, which was further confirmed with confocal microscopy analysis. Moreover, markedly higher bone marrow preservation was observed with histology in case of HAS supplementation compared to FCS. These findings indicate possible application of HAS in regenerative solutions of skeletal diseases like OA or osteonecrosis.

## 1. Introduction

Mesenchymal stem cells (MSCs) can be found in almost all tissues in the human body. They, being multipotent, can differentiate into multiple mature cell types like osteocytes, chondrocytes, or adipocytes under certain physiological or experimental conditions. Besides, they are able to replenish simultaneously the stem cell pool through self-renewal [1]. These features make them potential elements of cell-based therapies of the musculoskeletal system, for example, in case of osteoarthritis [2, 3], osteonecrosis [4], or other bone and cartilage injuries. The isolation method of this cell type is well described; therefore, it is an everyday process to extract MSCs from bone marrow, and a similar cell population can be obtained from adipose tissue, umbilical cord blood, or skeletal muscle [5–8]. As a drawback, during expansion in culture, animal sera like fetal calf serum (FCS) must be included as a supplement; otherwise, the cells stop proliferating. Bovine serum has economical, ethical, and scientific disadvantages such as immunogenicity, contaminations with prions and other pathogens, and the cost and ethical issues surrounding slaughtering calves just for their serum. These problems render animal serum a serious bottleneck of clinical applications [9–11]. However, it is unavoidable to supplement cell cultures with serum-specific growth factors, and other, still-unidentified factors of the serum as MSCs cannot survive in the absence of such components. Thus, human blood-derived additives like human serum albumin (HSA) [12] or platelet releasates such as platelet-rich plasma (PRP) are considered as efficient alternatives to replace FCS [13, 14]. The scientific rationale behind platelet-rich products is that thrombocytes provide a diverse growth factor supply such as platelet-derived growth factors (PDGFs), transforming growth factor beta-1 (TGF*β*-1) or vascular endothelial growth factor (VEGF), coagulation factors, serotonin, mitogens, and adhesion factors like fibronectin, fibrin, or vitronectin to support healing [15]. On the one hand, these molecules play a role in the proliferation and differentiation of expanded cells *in vitro* or during the early phases after implantation into the host. Besides, these same factors are crucial in the chemotaxis of helper cells like pluripotent stem cells from the ambient tissues [16]. In clinical indications, PRP is already widely used as a general “proliferative agent” in musculoskeletal diseases [17] such as knee osteoarthritis [18], rotator cuff pathology [19], osteonecrosis [20, 21], patellar tendinitis [22], muscle injury [23], and even total knee and hip replacements [24]. However, preparation protocols and nomenclature of these blood-derived products vary widely amongst authors and are often not well defined; therefore, results are difficult to reproduce and compare [25]. Additives like exogenous activators of the platelets (e.g., CaCl_2_ and bovine thrombin) can cause significant pain and burning sensation in the area of administration probably because of the pH alterations and the induced immune reactions of these additives [26, 27].

It is important to highlight that osteoarthritis (OA) is a prevalent disease with characteristic bone marrow lesions in the subchondral region [28, 29]. The loss of regenerative capacity of the cells in this location leads to bone cyst formation [30] and cartilage degeneration [31–33], which induces a chronic inflammatory cycle and breaks down the barrier of the joint space [34–36], inevitably destroying the joint. Regeneration of the healthy joint is mainly dependent on the subchondral bone marrow where MSCs are responsible for bone remodelling and the bottom-up restoration of the cartilage layer. An appropriate molecule cocktail could help the topical cells to overcome the degeneration and to regain their vitality at least to some extent which may kick-start the healing process.

Hyperacute serum (HAS) has been designed to avoid a number of disadvantageous effects of platelet releasates, since it works through natural coagulation in a single-step preparation process, avoiding issues with the overconcentrated plasma derivatives. Our research goal was to find cellular-level mode of action of HAS that is already being investigated for degenerative bone pathologies such as OA and osteonecrosis. Specifically, bone marrow lesions are observed in these pathologies due to the loss of regenerative capacity of the cells in this location. We set out to perform preclinical laboratory investigations on monolayer MSC cultures and in their natural niche, in a 3D subchondral bone marrow culture model (BMEs).

## 2. Materials and Methods

### 2.1. Blood Derivatives

Blood samples were obtained from 8 healthy donors, men and women (24–45 years), under IRB approval. The female donors were not pregnant and did not take medicine regularly, and they had no heavy diseases in the last two years (like myocardial infarction or cancer). Exclusion criterion was blood donation (over 250 mL) in the last six months. For HAS preparation, blood was collected in VACUETTE® 9 mL Z Serum C/A tubes (Greiner Bio-One, Austria) and centrifuged at 1710*g* for 5 minutes at room temperature. The fibrin clot formed in the tube was gently removed with a tweezer and placed onto a sterile Petri dish. With the use of a flat forceps, the serum portion was squeezed out of the fibrin clot, typically resulting in 1-2 mL HAS per tube. For PRP isolation, whole blood was obtained from donors in VACUETTE 9 mL K3 EDTA blood collection tubes (Greiner Bio-One, Austria) and centrifuged at 320*g* for 12 minutes at room temperature. The platelet-rich layer above the buffy coat was aspirated and transferred into a 15 mL tube and centrifuged at 1710*g* for 10 min. The resulting platelet pellet was resuspended in the same volume as the isolated HAS from the same donor in order to maintain comparability at the donor level (Supplementary Figure 1). Blood derivatives were either used fresh or stored at −20°C until use. All HAS and PRP samples were pooled samples containing at least two donor's blood sample. In order to determine the blood derivatives' platelet, white blood cell, red blood cell, and growth factor, profile automated cell counter, Proteome Profiler Assay, and LUMINEX Assay were used, and data are published in an earlier publication [37].

### 2.2. Cell Culture

All cell culture procedures were carried out in a sterile laminar flow tissue culture hood. Cells were maintained in an incubator at 37°C and 5% CO_2_ at 95% humidity. Human mesenchymal stem cells (MSCs) were purchased from Lonza (Walkersville, USA) and ATCC (Manassas, USA). The experiments were performed with at least 2 batches of MSCs. The cells were seeded at 5000 cells/cm^2^ in T-75 tissue culture flasks and maintained in standard growth medium. The growth medium was composed of Dulbecco's modified Eagle's medium (DMEM), 4,5 g/L glucose, GlutaMAX™ Supplement, and pyruvate (Gibco, Paisley, Scotland), supplemented with 10% (v/v) fetal calf serum (FCS, Gibco, Paisley, Scotland), basic fibroblast growth factor (bFGF) at 1 ng/mL (Sigma-Aldrich, St. Louis, USA), 2% penicillin/streptomycin (Sigma-Aldrich, St. Louis, USA), and 1% amphotericin (Sigma-Aldrich, St. Louis, USA). Cell culture medium was refreshed twice a week.

### 2.3. Cell Proliferation Measurements of MSCs

MSCs between passages 3 and 9 were seeded in standard growth medium in 5 parallel wells of a 96-well plate (2000 cells/well). Forty-eight hours after the start of the incubation, on day zero, standard growth medium called “FCS” (hereafter) was refreshed only in the control wells; in the others, it was changed with 10% (v/v) FCS + 1 ng/mL bFGF in “FCS + bFGF” group (hereafter), or 10% (v/v) PRP in “PRP” group (hereafter), or 10% (v/v) HAS in “HAS” group (hereafter). The PRP-supplemented medium contained 2 U/mL heparin (Clexane, Sanofi Aventis, Paris, France) in order to prevent clotting in the culture medium. As negative control, serum-free medium was used (“serum-free” group hereafter). Cell-free wells were used as a technical background. Cell viability at 0, 2, and 5 days in culture was determined using Cell Proliferation Kit II (XTT; Roche, Mannheim, Germany) according to the manufacturer's instructions. Absorbance was measured after 4 hours' incubation in the staining solution using a PowerWave microplate spectrophotometer (BioTek, Winooski, VT, USA) at 480 nm with a reference wavelength at 650 nm. The experiments were replicated three times.

### 2.4. Isolation, Culture, and Viability Measurement of Bone and Marrow Explants (BMEs)

**Figure 1:**
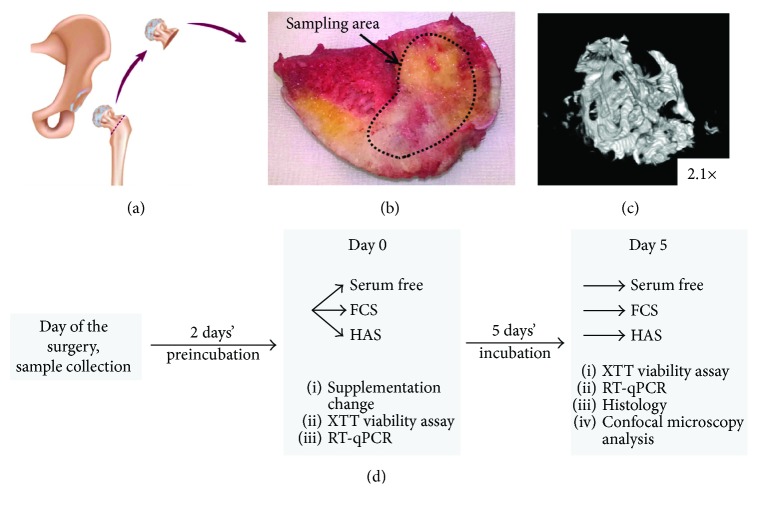
Bone marrow tissue explantation model. During total hip replacement surgery, the femoral head was discarded (a) and replaced with an endoprosthesis. For experimental reasons, small bone pieces from the cut surface of the femoral head were isolated with a sharp chisel (b). *μ*CT image shows the structure of the BMEs (c). (d) The experimental setup of the BMEs' and MSC cell cultures.

BMEs of approximately 2 mm in diameter were harvested from patients undergoing routine total hip replacement surgery at the Department of Orthopedics, Semmelweis University (Budapest, Hungary). All patients were more than 40 years old and diagnosed with osteoarthritis. The femoral heads that would have otherwise been discarded were used for BME harvesting under IRB approval. The femoral head was cut in half with a wide bone chisel, and BMEs were harvested from the cut surface with a small chisel (Figure 1). Explants were delivered to the laboratory in the standard growth medium and incubated under standard cell culture conditions for 48 hours before any further experiments. BMEs were treated with the same media as MSCs, that is, FCS, HAS, and serum-free media. PRP was not used as serum supplement in case of BMEs, because of its special effect experienced in MSCs' culture. Its unique influence on BMEs in comparison with HAS is also intriguing, and a separate study may summarize it. For XTT viability measurements, bone pieces were transferred into a new plate immediately before measurement in order to exclude cells that may have grown out from the tissue onto the plastic surfaces. The protocol was the same as in the case of MSCs. Results were normalized with the dry weight of the BMEs considering that it is proportional to the number of active cells.

### 2.5. Flow Cytometry

Flow cytometry with lineage-specific markers (Beckman Coulter, CA, USA) was performed to detect lineage shift in the presence of various blood derivatives. According to the manufacturer's recommendation, only cells between passages 3 and 9 were used for the experiments. The same amount (250000 cells per flask) of MSCs were seeded in four T-25 flasks and cultured for 48 hours with standard growth medium. After this preincubation, old media were discarded, and new media were added containing FCS, FCS + bFGF (the medium recommended by the manufacturer, which warrants the MSC phenotype), PRP, or HAS as supplement. Media were exchanged every 48 hours, until cells in one or more groups reached 100% confluency (5 days after special media were added). Cells were detached with Accutase (Sigma-Aldrich, St. Louis, USA), centrifuged two times (10 min, 500*g*) in FACS buffer (1% [v/v] FCS in phosphate-buffered saline [PBS]), and half of each cell culture pool was stained with antibodies, as described in Supplementary Table 2 (Human MSC Analysis Kit, BD Biosciences, St. Louis, USA). The other unlabelled half of the pool was used as control isotypic antibodies to exclude nonspecific staining.

### 2.6. RT-qPCR

Total RNA from MSCs was isolated by using High Pure RNA Isolation Kit (Roche Diagnostics GmbH, Mannheim, Germany). Experiments were replicated five times. For RNA isolation from BMEs, 4 or 5 frozen bone chips from the same patient and from the same treatment were homogenized in a mortar in liquid nitrogen. Four patient's samples were used for this evaluation. The tissue slush was lysed in TRIzol Reagent (Invitrogen, CA, USA), centrifuged once at 12000*g* for 30 seconds, and the supernatant was cleaned with Direct-zol™ RNA MiniPrep Kit (Zymo Research CA, USA). RNA yield was determined using a NanoDrop 1000A spectrophotometer (Thermo Fisher Scientific, Waltham, MA, USA). The quality of RNA extract was verified based on the generally used ratio of absorbance at 260/280 nm and 260/230 nm, and by electrophoretogram of the samples on 1% agarose gel. The isolated RNA was reverse transcribed using ReadyScript™ reverse transcription kit (Sigma-Aldrich, St. Louis, USA) primed by an oligo(dT) primer according to the manufacturer's instructions. TaqMan Gene Expression Assays were purchased from Thermo Fisher Scientific Inc., and real-time quantitative polymerase chain reaction (RT-qPCR) was performed with an Applied Biosystems 7500 real-time PCR system. Details of the TaqMan assays are listed in Supplementary Table 1. The ΔΔC_T_ method was used for quantification, where the threshold cycle values were normalized to the corresponding one of actin beta mRNA and viability of the samples. Experimental times were scheduled in the same way as it was in case of viability tests; namely, RNA was isolated on day 0 and after 5 days' incubation in FCS, FCS + bFGF, PRP, or HAS medium. In case of BMEs, the results are expressed as fold values compared to those of the starting day of the experiments and multiplied with the actual viability showing the net increase of the expression level.

### 2.7. Confocal Microscopy of BMEs

#### 2.7.1. Live/Dead Staining

After incubation of BMEs in different media, bone chips were washed three times with PBS and stained in PBS containing 1 *μ*M Calcein-AM (Invitrogen, Carlsbad, CA, USA), 5 *μ*g/mL ethidium homodimer (Invitrogen, Carlsbad, CA, USA), and 5 *μ*g/mL Hoechst 33342 (Invitrogen, Carlsbad, CA, USA) for 40 minutes. The experiments were performed in case of two patients' four-four BMEs (8 replica) after 5 days' incubation in FCS, HAS, or serum-free media, in total 3 different media. The samples were washed three times with PBS and imaged immediately with a Nikon A1R confocal microscope (Nikon-KOKI Imaging Center, Budapest).

#### 2.7.2. Immunostaining

BMEs were fixed in 4% paraformaldehyde for 40 minutes at room temperature and stained with Human Mesenchymal Stem Cell Kit's anti-CD19 and anti-CD44 component (Merck Millipore, Germany) according to the manufacturer's instructions. The experiments were performed in case of two patients' four-four BMEs after 5 days' incubation in FCS, HAS, or serum-free media. Samples were imaged in PBS buffer in glass bottom dishes using a Nikon Ti A1R confocal scanning microscope with 4x and 10x objective (Plan Fluor, NA = 0.3).

### 2.8. Histology

BMEs were fixed in 4% formalin and dehydrated in an ascending alcohol series at room temperature, embedded in a special resin developed for mineralized tissues (Technovit 9100, Kulzer), and decalcified. Sections of 6–8 *μ*m were cut using Leica RM2255 sawing microtome and stained with hematoxylin and eosin (H&E), von Kossa (VK), and Masson's trichrome (MT). Three bone biopsies were taken from 3 different patients (at the same day); each contained several BMEs, enough for all planned conditions. Three BMEs of each patients were fixed on day 0 (in FCS buffer, used as control) and the remaining 3-3 BMEs following 5 days' incubation further in FCS buffer or in HAS medium as replacement for FCS buffer, leading in total to 3 treatment groups. Three-three replicates of one type of treatment group but from different patients were finally mixed and then analyzed regardless of the origin (patient), to get statistically acceptable results. For the evaluation of the slides, Nikon Eclipse 80i light microscope was used. Quantification of the percentage of osteoid tissue on bone lamellae surface was performed using ImageJ software and was determined as area of the osteoid tissue section/(area of the mineralized bone lamellae section × area of the osteoid tissue section). Histological scores for the bone marrow integrity analysis were tested by 4 persons; the possible scores were as follows: no valuable bone marrow structure = 0, minimal level of bone marrow structure = 1, average level of bone marrow structure = 2, and high level of bone marrow structure = 3.

### 2.9. Statistical Analysis

One-way analysis of variance (ANOVA) with Tukey post hoc analysis was used to compare means of experimental groups using a significance level of *p* > 0.05. Analysis was performed using Prism software. Data are presented as mean ± SEM.

## 3. Results

### 3.1. Mesenchymal Stem Cells (MSCs) Proliferate Intensely in HAS-Supplemented Cell Culture

MSCs form the basis of the regenerative capacity of the osteochondral niche. We tested the effects of serum derivatives on these cells *in vitro.* Subconfluent MSC cultures were incubated for 2 or 5 days in serum-free DMEM medium, or medium supplemented with FCS (standard constituent of stem cell media), FCS + bFGF, PRP, or HAS, 10% (v/v) each. Viability of the samples was measured with XTT assay on the 1st, 2nd, and 5th days of the experiment.

**Figure 2:**
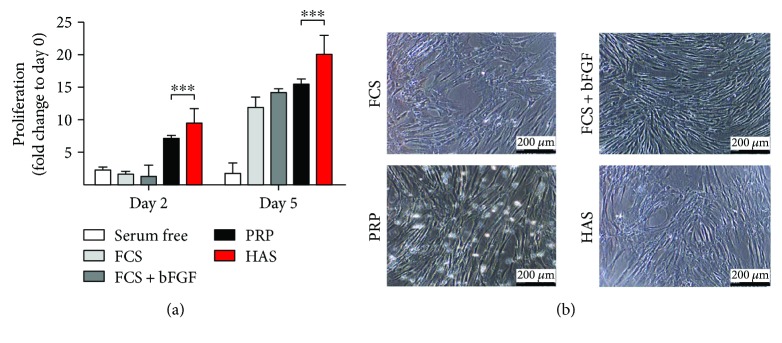
Time-course effect of serum supplements on MSCs. (a) Subconfluent MSCs were cultured in DMEM in the absence of supplement (serum free (white bar)), or supplemented with FCS (light-gray bar), FCS + bFGF (dark-gray bar), PRP (black bar), or HAS (red bar). (b) Cell morphology of MSCs using phase-contrast microscopy. ^∗∗∗^Confidence interval *P*-value: 0.0001 to 0.001, extremely significant.

As shown in Figure 2(a), SF, FCS, and FCS + bFGF had no mitogenic effect after 2 days of incubation. In the presence of PRP and HAS, viability of cells was elevated 7.18 ± 0.18-fold and 9.57 ± 0.94-fold, respectively.

In the period between the 2nd and 5th days, FCS + bFGF caused an intense (14.18 ± 0.56-fold) cell number elevation. FCS and PRP reached similar viability to that of FCS + bFGF for the 5th day, 11.84 ± 0.74-fold and 15.54 ± 0.33-fold, respectively. HAS had the strongest effect as supplement in the cell culture medium, whereby the cell viability was 20.11 ± 1.43-fold higher compared to that at the starting day (day 0). This effect is very remarkable considering that the widely used culture medium and the treatment with a growth factor were less effective (Figure 2(a)).

The morphology of the cells was not visibly altered by the various treatments; all preserved the typical MSC morphology (Figure 2(b)).

### 3.2. MSC Markers Are Retained When Cells Are Cultured in HAS-Supplemented Medium

**Figure 3:**
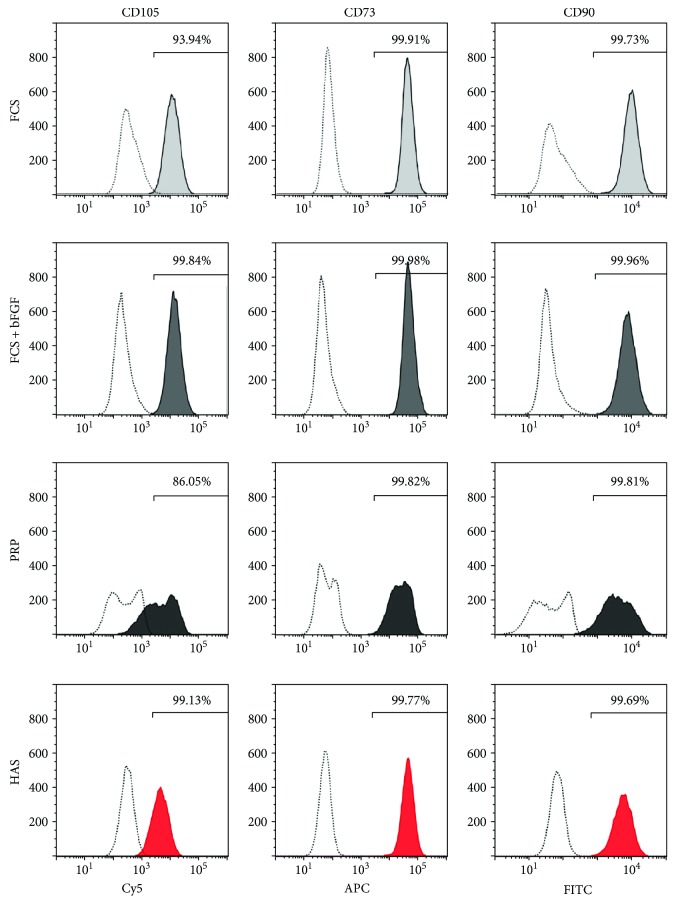
Cell immunophenotypes of MSCs cultured in differently supplemented media. MSCs were cultured in FCS (light-gray shading), FCS + bFGF (dark-gray shading), PRP (black shading), or HAS (red shading); stained with specific antibodies; and analyzed with flow cytometry. Unstained control cells are represented by dotted lines. Representative images of MSC and hematopoietic marker expression.

Since faster proliferation may lead to lineage shift, immunophenotyping analysis was performed to characterize that MSCs cultured for 5 days in differently supplemented media preserved their MSC characteristics. MSCs cultured in FCS, FCS + bFGF, or HAS medium were positive for CD90-FITC, CD105-PerCP-Cy5.5, and CD73-APC in more than 93.94%. While CD90-FITC and CD73-APC expression was above 99% in case of the PRP-supplemented samples, CD105-PerCP-Cy5.5 level decreased by 13.95%, and two distinct peaks appeared; however, both peaks still fell into the desired ranges. This finding indicates that MSCs in PRP may have already started to vary in some aspects in the early phase of incubation (Figure 3). This was not observed with FCS or HAS treatments.

### 3.3. MSC-Specific Gene Expression Does Not Change in Case of HAS Supplementation

**Figure 4:**
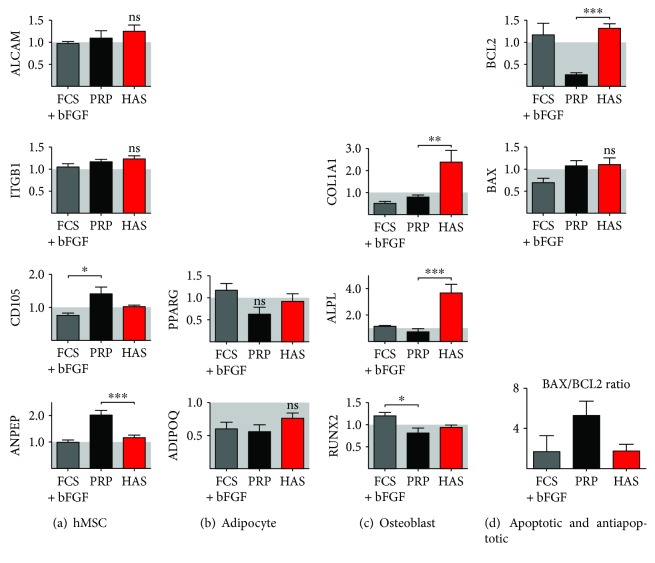
Gene expression analysis of MSCs cultured in serum-supplemented media. Following 5 days' culture in FCS + bFGF (gray bar), PRP (black bar), or HAS (red bar) and FCS as control, mRNA was isolated for RT-qPCR analysis of MSC- (a), adipocyte- (b), osteoblast- (c), apoptosis-, and antiapoptosis-specific (d) genes. Data are presented as fold change values to the expression of MSCs cultured in FCS-supplemented medium that was considered as the standard growth medium. ^∗^Confidence interval *P*-value 0.05 to 0.01, significant; ^∗∗^ *P*-value 0.001 to 0.01, very significant; ^∗∗∗^ *P*-value 0.0001 to 0.001, extremely significant.

The potential lineage-specific changes were screened with gene expression analysis. The expression of MSC-specific genes was confirmed by real-time qPCR after 5 days' incubation in FCS-, FCS + bFGF-, PRP-, and HAS-supplemented media. The results are presented as fold values compared to those of the medium recommended for culture by the manufacturer, which contains FCS and preserves totally the mesenchymal stem cell surface markers. ALCAM/CD166, ITGB1, ENG/CD105, and ANPEP expression was retained and slightly elevated in the HAS-supplemented samples when compared to that of the FCS-supplemented group by 1.25 ± 0.13-fold, 1.24 ± 0.06-fold, 1.03 ± 0.03-fold, and 1.16 ± 0.09-fold, respectively. While FCS + bFGF had no effect (0.96 ± 0.05-fold, 1.06 ± 0.063-fold, 0.77 ± 0.05-fold, and 0.98 ± 0.08-fold, resp.) on the expression of the same markers, in case of the PRP-treated cells, remarkable increase was observed: 1.09 ± 0.16-fold, 1.18 ± 0.04-fold, 1.42 ± 0.19-fold, and 2.01 ± 0.17-fold, respectively (Figure 4(a)).

### 3.4. HAS Supplementation Induced Osteogenic but Not Adipogenic Differentiation in MSC Culture

Since MSCs are the common progenitor cells of adipocytes and osteoblasts, adipocyte-specific and osteoblast-specific gene expression was investigated in the variously supplemented MSC cultures by real-time qPCR. PPARG and ADIPOQ expression, markers of adipogenic differentiation, was retained or decreased when FCS supplementation was changed to FCS + bFGF, PRP, or HAS (Figure 4(b)), indicating that the cells are not differentiating into these lineages. COL1A1, ALPL, and RUNX2 are characteristic of osteoblastic differentiation. The expression of these markers was slightly changed in cultures supplemented with FCS + bFGF (0.51 ± 0.08-fold, 1.14 ± 0.04-fold, and 1.2 ± 0.07-fold, resp.). PRP had the same effect: 0.79 ± 0.8-fold change in COL1A1 expression, 0.75 ± 0.2-fold change in ALPL expression, and 0.81 ± 0.11-fold change in RUNX2 expression compared to those of the FCS-supplemented group.

Interestingly, HAS supplementation resulted in a significant increase of osteoblastic gene expression. COL1A1 mRNA level elevated 2.38 ± 0.51-fold, ALPL level 3.68 ± 0.65-fold, and RUNX2 level 0.94 ± 0.04-fold compared to those of the FCS-supplemented group (Figure 4(c)). This pattern of gene expression, together with the proliferation, phenotype, and CD marker data, suggests that HAS is the most proliferative factor for MSCs, outperforming even PRP despite the fact that PRP has much higher amounts of growth factors. The phenotype of MSCs is largely unaffected by the treatments, with a trend towards more osteogenic lineage in case of the presence of HAS.

### 3.5. BAX/BCL2 Ratio Was Highly Increased When MSC Culture Was Supplemented with PRP

BAX and BCL2 are two members of a gene family involved in the regulation of cellular apoptosis. BAX is characterized as an apoptosis-promoting factor while BCL2 as an apoptosis-suppressing factor. The cells with a high BAX/BCL2 ratio are more sensitive to the apoptotic stimuli than are those with low BAX/BCL2 ratio. In case of MSCs, BAX/BCL2 ratio decreased by FCS + bFGF supplementation by 1.74 ± 1.5-fold in comparison to FCS supplementation, and also, HAS supplementation did not cause remarkable change in this value (1.72 ± 0.69-fold). In case of PRP supplement, this ratio elevated to 5.27 ± 1.44-fold of the normal value (Figure 4(d)), which indicates important increase of cellular sensitivity to apoptosis.

### 3.6. HAS Promotes Cell Viability of Bone Marrow Explant (BME) Cells

**Figure 5:**
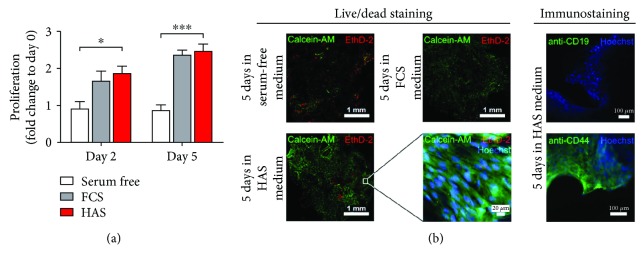
Culture of BMEs. Viability of BMEs cultured in serum-supplemented media (a). Serum-free medium (white bard) and medium supplemented with FCS (gray bar) or HAS (red bar). (b) Representative confocal microscopy images stained with the live/dead dye combination Calcein-AM/ethidium homodimer. Nuclei were counterstained with Hoechst. Coloured terms indicate the colour of the representative dye. ^∗^Confidence interval *P*-value 0.05 to 0.01, significant; ^∗∗∗^ *P*-value 0.0001 to 0.001, extremely significant.

For a better understanding, we continued our experiments at the tissue level, in freshly harvested human osteoarthritic bone and marrow explants, as its clinical situation is closest to that of a bone marrow lesion. BMEs were isolated from osteoarthritic subchondral space-femoral heads discarded at hip replacement operations and maintained under normal cell culture conditions, and supplementation of the media with either FCS or HAS was required for cell growth; cell proliferation was investigated in a similar 5-day time frame on BMEs as in MSCs. Hence, the size of the bone pieces was slightly different, and the measured absorbance values were normalized with the dry weight of the BMEs.  Figure 5(a) shows that the serum-free culture environment, as it was expected, did not promote cell proliferation neither for the second day nor for the 5th day. However, the viability of the BMEs elevated significantly for both the second and 5th days in case of FCS (1.66 ± 0.26-fold and 2.36 ± 0.12-fold, resp.) and HAS supplementation (1.87 ± 0.18-fold and 2.46 ± 0.19-fold, resp.) (Figure 5(a)). We visualised with confocal microscopy the cells on the surface of bone lamellae. In serum-free environment after 5 days' incubation, a very low amount of living cells were detectable, green-labelled cell cytoplasms that were not that much observable. Some red areas show dead cells on the surface. In case of FCS and HAS, at 5-day-long supplementation, a high amount of living cells were observable on the surface of the bone lamellae. Immunostaining of the HAS-supplemented samples was performed with anti-CD19 antibody for detection of hematopoietic stem cells on the BMEs' surface. Hoechst nucleus staining showed a high number of living cells that were not CD19 positive (green); that is, they were not hematopoietic stem cells. Anti-CD44 staining detected living MSCs on the bone lamellae.

### 3.7. HAS Initiates Osteogenic Differentiation in BMEs

**Figure 6:**
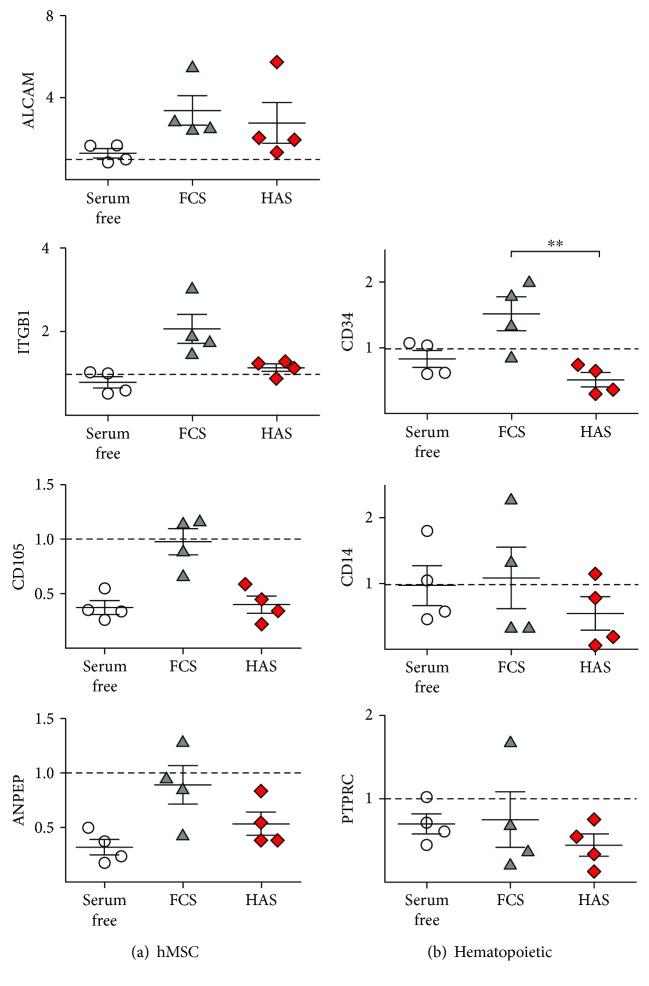
MSC-specific and hematopoietic stem cell-specific gene expression profiles of BMEs as determined by RT-qPCR. Total RNA extraction was performed from BMEs cultured either in serum-free medium (white circles) or in medium supplemented with FCS (gray triangles) or HAS (red diamonds) after 5 days' incubation. ^∗∗^Confidence interval *P*-value 0.001 to 0.01, very significant.

We compared gene expression of BMEs cultured within serum-free medium, FCS, or HAS. A set of characteristic mesenchymal markers was analyzed after 5 days of incubation. As shown in Figure 6(a), MSC markers' mRNA level was retained or elevated in culture of FCS (ALCAM/CD166: 3.37 ± 0.72-fold; ITGB1: 2.06 ± 0.34-fold; ENG/CD105: 0.97 ± 0.11-fold; ANPEP: 0.89 ± 0.17-fold) and stayed unchanged or decreased in HAS (ALCAM/CD166: 2.76 ± 1.00-fold; ITGB1: 1.14 ± 0.08-fold; ENG/CD105: 0.39 ± 0.07-fold; ANPEP: 0.53 ± 0.11-fold, resp.) for the 5th day in comparison to the day 0 values.

We analyzed the expression of a wide panel of characteristic hematopoietic stem cell (HSC) markers to examine the opportunity of proliferation of resident HSCs. As shown in Figure 6(b), CD34, CD14, and PTPRC expression was unchanged or increased in FCS (1.51 ± 0.25-fold, 1.09 ± 0.47-fold, and 0.75 ± 0.32-fold, resp.) but markedly decreased in HAS (0.51 ± 0.25-fold, 0.54 ± 0.25-fold, and 0.44 ± 0.13-fold, resp.). These observations support that findings that in HAS-supplemented environment, the not-grown HSCs provide the increase of measured viability values.

**Figure 7:**
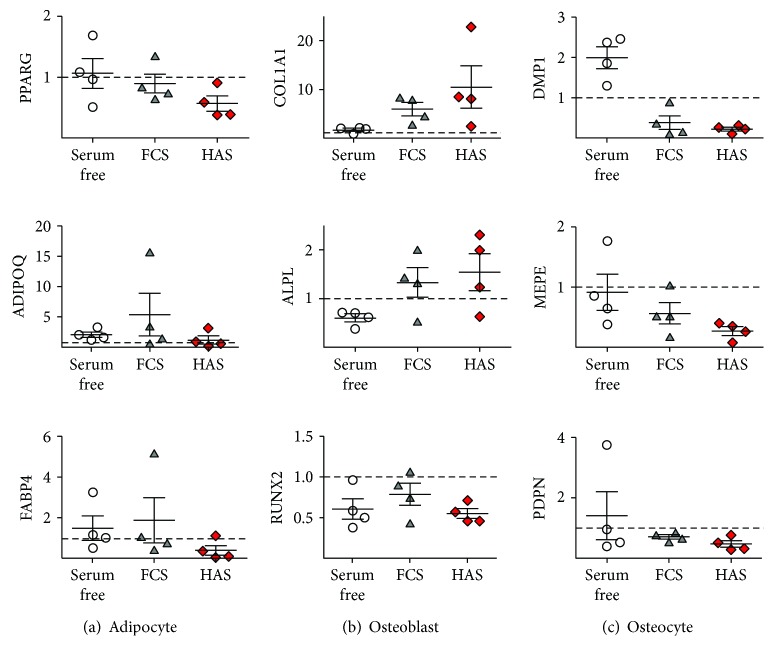
Adipocyte-, osteoblast-, and osteocyte-specific gene expression profiles of BMEs as determined by RT-qPCR. Total RNA extraction was performed from BMEs cultured either in serum-free medium (white circles) or in medium supplemented with FCS (gray triangles) or HAS (red diamonds) after 5 days' incubation.

MSCs are the common progenitor cells of adipocytes and osteoblasts; therefore, characteristic genes' expression of these two lineage was examined as well. PPARG, ADIPOQ, and FABP4, the adipocyte-related genes, were mostly downregulated or unchanged in all culture environment (0.89 ± 0.15-fold, 5.36 ± 3.52-fold, and 1.85 ± 1.11-fold, resp., in FCS; and 0.56 ± 0.12-fold, 1.17 ± 0.65-fold, and 0.38 ± 0.24-fold, resp., in HAS) (Figure 7(a)). However, COL1A1 and ALPL, the specific osteoblast markers, showed a trend towards upregulation at the 5-day time point (5.92 ± 1.34-fold and 1.33 ± 0.31-fold, in FCS, resp.; 10.42 ± 4.35-fold and 1.54 ± 0.37-fold, in HAS, resp.) (Figure 7(b)). Interestingly, RUNX2, one of the most examined osteoblast differentiation-related markers, did not increase (0.79 ± 0.13-fold in FCS and 0.55 ± 0.06-fold in HAS) (Figure 7(b)). Characteristic osteocyte-specific genes were analyzed, as well as DMP1, MEPE, and PDPN; however, according to the results, this cell type did not survive the applied culturing conditions (0.38 ± 0.17-fold, 0.56 ± 0.17-fold, and 0.71 ± 0.06-fold, resp., in FCS; 0.22 ± 0.04-fold, 0.27 ± 0.07-fold, and 0.47 ± 0.11-fold, resp., in HAS) (Figure 7(c)).

### 3.8. More Preserved Bone Marrow Integrity Was Found in Case of HAS Supplementation of BMEs

**Figure 8:**
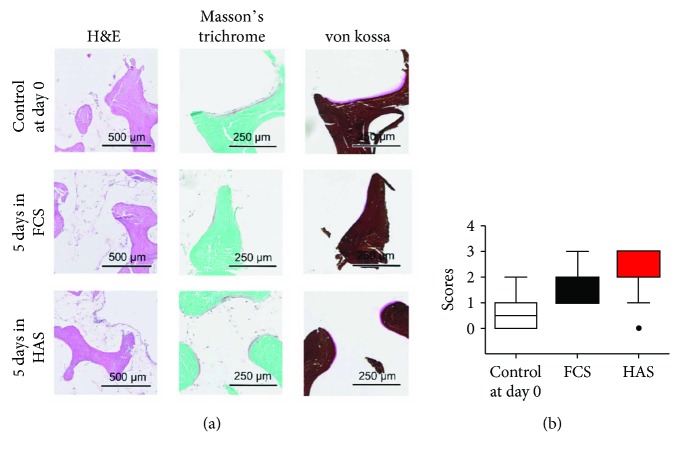
Histological analysis of BMEs. (a) Representative images of HAS- and FCS-supplemented samples stained with hematoxylin and eosin, Masson's trichrome, and von Kossa. (b) Histological scores of bone marrow integrity.

Culturing BMEs in HAS-supplemented medium for 5 days preserved bone marrow integrity as representative hematoxylin and eosin-stained sections show. FCS supplementation for 5 days appeared less effective therein (Figure 8(a)). We evaluated with histological scores of the bone marrow integrity in case of the control samples (day 0) and after 5 days' incubation in FCS- or HAS-supplemented medium. HAS supplementation showed obvious increase in marrow structure preservation or formation as did the other supplementation (Figure 8(b)).

The quantification of the percentage of osteoid tissue on bone lamellae showed less decrease in case of HAS supplementation as in case of FCS supplementation of the culture medium compared to that in the control day (day 0), when the experiment started (control day: 0.0047 ± 0.0010%; 5 days in FCS: 0.0025 ± 0.0003%; 5 days in HAS: 0.0037 ± 0.0006%).

## 4. Discussion

Hyperacute serum (HAS) extraction protocol was designed to be as close to the physiological activation of blood upon injury as technically possible. The resulting serum is free of cells, platelets, fibrin, anticoagulants, and exogenous activation substances and represents the extracellular matrix milieu immediately after injury and blood clotting [37]. Consequently, it avoids the disadvantages of activated and enriched blood plasma varieties and the more complex preparation process they require. On the other hand, since HAS contains physiological concentrations of growth factors and cytokines, which may be diluted at the place of application, enriched preparations may have an advantage depending on local factors at the host tissue. Interestingly, in the current study, we have observed that the most proliferative cell culture milieu was provided by HAS supplementation, and not with PRP, despite the higher number of growth factors in the latter. One explanation can be that the cells respond best to optimized conditions, and any deviation from this, either too little or too much paracrine signals, turn into inhibition or act against each other on the same cell. HAS supports the proliferation of MSCs in monolayer; moreover, the influence on proliferation outperforms that of the standard culture conditions (FCS or FCS + bFGF) or that of PRP-supplemented media. The positive changes foreshadow the effective future application in ex vivo expansion of bone marrow or other human tissue-derived stem cells before administration in diverse cell therapies. According to our flow cytometry analysis, MSC characteristics of the cultivated cells were retained in case of all supplement types; however, PRP-supplemented cells showed two distinctive peaks that indicate a change in their phenotype. This supplement leads to increased BAX/BCL2 ratio on mRNA level, which presumably implies disadvantageous effects as is related to initiation of apoptotic processes. During a cell transplantation procedure, a live but apoptotic cell mass is useless in terms of expected therapeutic effects. Thus, the use of PRP does not seem to be the optimal choice for cell therapy supplementation due to the overdose of introduced cytokines on the one hand and the proapoptotic effect on the other.

We have found that gene expression analysis following the treatment of MSCs with HAS showed significant increase in the expression of osteoblast markers COL1A1 and ALPL. This raises the possibility of the medical use of this HAS supplementation in musculoskeletal diseases, where impaired bone metabolism is observable such as in osteoarthritis (OA) or osteonecrosis. In OA, the regeneration of the healthy joint is mainly dependent on the subchondral bone marrow where MSCs are responsible for bone remodelling and the bottom-up restoration of the cartilage layer. Therefore, it can be hypothesized that restoring the regenerative capacity of subchondral bone marrow may hinder the progression of OA. For the better understanding of this process, we used an *in vitro* 3D model, namely, human subchondral osteoarthritic bone pieces (BMEs) cultured with various serum supplements. The positive influence of HAS on the proliferation of resident MSCs on the surface of the bone lamellae has been proven by viability tests and fluorescence microscopy analysis, basically reproducing the data obtained in monolayer in a complete bone tissue. Similarly, the gene expression pattern of the mixed cell population of the explants shows a relative increase of MSC and osteoblast markers (COL1A1 and ALPL). In addition, osteoblast and osteoarthritic chondrocyte proliferation rates were higher in HAS-supplemented media compared to PRP, indicating that the positive effects are also observed on other musculoskeletal cell types (Supplementary Figures 2 and 3). This has been also shown in histology analysis where more preserved bone marrow structure was demonstrated after HAS treatment.

## 5. Conclusion

Despite their seemingly similar preparation methods, serum derivatives can have very divergent biological effects as it was demonstrated here between hyperacute serum and PRP. Taken together, the five separate lines of experimental observations regarding the effect of hyperacute serum point in one direction—(1) MSCs proliferate better, (2) MSCs shift lineage towards the osteoblastic line, (3) osteoblasts proliferate faster, (4) chondrocytes proliferate faster even when affected by osteoarthritis, and (5) bone marrow structure—is more preserved: hyperacute serum may be applicable in therapeutic protocols targeting the degenerative bone marrow niche.

## Supplementary Materials

Supplementary Materials Supplementary Figure 1: isolation method of HAS and PRP. Supplementary Table 1: real-time qPCR primer collection. Supplementary Table 2: flow cytometry antibody cocktails. Supplementary Figure 2: proliferation effect of serum supplements on osteoblast culture. Subconfluent osteoblasts (Lonza, Walkersville, USA) were cultured in high-glucose DMEM with pyruvate (Gibco, Paisley, Scotland) and with antibiotics (penicillin 200 U/mL, streptomycin 0.2 mg/mL, and amphotericin B 2.5 *μ*g/mL) (Sigma-Aldrich, St. Louis, USA) and L-ascorbic acid (50 *μ*g/mL; Sigma-Aldrich, St. Louis, USA) in the absence of supplement (white bar), 10 (v/v)% of FCS (light-gray bar), 10 (v/v)% FCS + 1 ng/mL bFGF (dark-gray bar), 10 (v/v)% PRP (black bar), or 10 (v/v)% HAS (red bar). XTT viability assay was performed on the third, 11th, and 18th days. Results are presented as means of triplicate samples in three separate cultures (*n* = 9 altogether). Supplementary Figure 3: proliferation effect of serum supplements on osteoarthritic chondrocytes. Cells were cultured in DMEM/F12 (Gibco, Paisley, Scotland) with antibiotics (penicillin 200 U/mL, streptomycin 0.2 mg/mL, and amphotericin B 2.5 *μ*g/mL) (Sigma-Aldrich, St. Louis, USA), and L-ascorbic acid (50 *μ*g/mL; Sigma-Aldrich, St. Louis, USA), in the absence of supplement (white bar), 10 (v/v)% of FCS (light-gray bar), 10 (v/v)% FCS + 1 ng/mL bFGF (dark-gray bar), 10 (v/v)% PRP (black bar), or 10 (v/v)% HAS (red bar). XTT viability assay was performed on the 4th and 8th days. Results are presented as means of triplicate samples in three separate cultures (*n* = 9 altogether).
