# Supplementary material for: The Effects of Hyperacute Serum on the Elements of the Human Subchondral Bone Marrow Niche
Source: Stem Cells Int. 2018 Apr 16;2018:4854619. doi: 10.1155/2018/4854619 (PMC5926480; doi:10.1155/2018/4854619)
Supplement: Supplementary Materials — Supplementary Figure 1: isolation method of HAS and PRP. Supplementary Table 1: real-time qPCR primer collection. Supplementary Table 2: flow cytometry antibody cocktails. Supplementary Figure 2: proliferation effect of serum supplements on osteoblast culture. Subconfluent osteoblasts (Lonza, Walkersville, USA) were cultured in high-glucose DMEM with pyruvate (Gibco, Paisley, Scotland) and with antibiotics (penicillin 200 U/mL, streptomycin 0.2 mg/mL, and amphotericin B 2.5 μg/mL) (Sigma-Aldrich, St. Louis, USA) and L-ascorbic acid (50 μg/mL; Sigma-Aldrich, St. Louis, USA) in the absence of supplement (white bar), 10 (v/v)% of FCS (light-gray bar), 10 (v/v)% FCS + 1 ng/mL bFGF (dark-gray bar), 10 (v/v)% PRP (black bar), or 10 (v/v)% HAS (red bar). XTT viability assay was performed on the third, 11th, and 18th days. Results are presented as means of triplicate samples in three separate cultures (n = 9 altogether). Supplementary Figure 3: proliferation effect of serum supplements on osteoarthritic chondrocytes. Cells were cultured in DMEM/F12 (Gibco, Paisley, Scotland) with antibiotics (penicillin 200 U/mL, streptomycin 0.2 mg/mL, and amphotericin B 2.5 μg/mL) (Sigma-Aldrich, St. Louis, USA), and L-ascorbic acid (50 μg/mL; Sigma-Aldrich, St. Louis, USA), in the absence of supplement (white bar), 10 (v/v)% of FCS (light-gray bar), 10 (v/v)% FCS + 1 ng/mL bFGF (dark-gray bar), 10 (v/v)% PRP (black bar), or 10 (v/v)% HAS (red bar). XTT viability assay was performed on the 4th and 8th days. Results are presented as means of triplicate samples in three separate cultures (n = 9 altogether). [file 4854619.f1.pdf]

Supplementary data

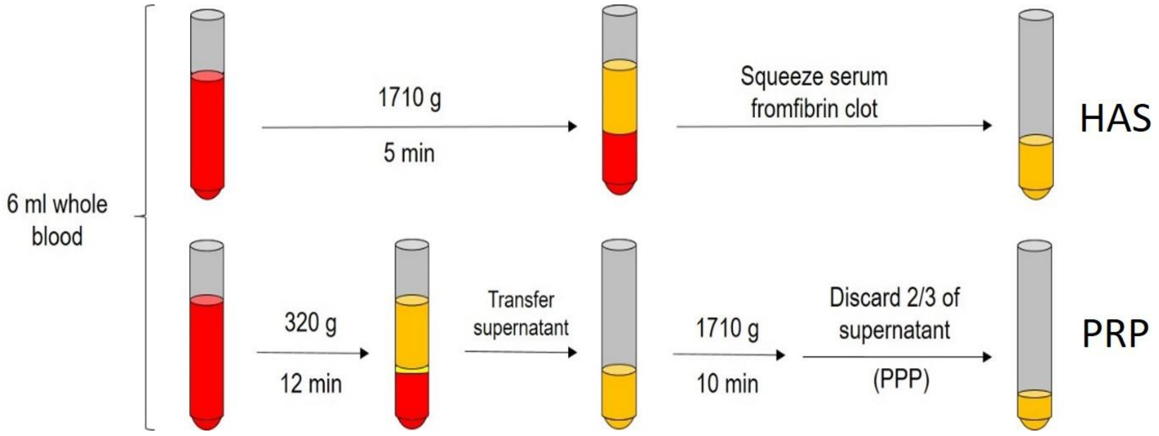

Supplementary figure 1. **Isolation method of HAS and PRP.**

		GENE NAME	ASSAY ID	GENE SYMBOL	ENTREZ GENE ID
1.	Mesenchymal stem cell markers	activated leukocyte cell adhesion molecule	Hs00977641_m1	ALCAM	214
2.		integrin subunit beta 1	Hs01127536_m1	ITGB1	3688
3.		alanyl aminopeptidase	Hs00174265_m1	ANPEP	290
4.		endoglin	Hs00923996_m1	ENG	2022
5.	Osteoblast markers	collagen type I.	Hs00164004_m1	COL1A1	1277
6.		runt related transcription factor 2	Hs01047973_m1	RUNX2	860
7.		alkaline phosphatase, liver/bone/kidney	Hs01029144_m1	ALPL	249
9.	Adipocyte markers	peroxisome proliferator activated receptor gamma	Hs00234592_m1	PPARG	5468
10.		fatty acid binding protein 4	Hs01086177_m1	FABP4	2167
11.		adiponectin	Hs00605917_m1	ADIPOQ	9370
12.	Hematopoietic stem cell markers	CD14	Hs02621496_s1	CD14	929
13.		CD34	Hs02576480_m1	CD34	947
14.		protein tyrosine phosphatase, receptor type C	Hs04189704_m1		5788
15.	Osteocyte markers	podoplanin	Hs00366766_m1	PDPN	10630
16.		dentin matrix acidic phosphoprotein 1	Hs01009390_m1	DMP1	1758
17.		matrix extracellular phosphoglycoprotein	Hs00220237_m1	MEPE	56955
18.	Apoptotic marker	BCL2 associated X	Hs00180269_m1	BAX	581
19.	Antiapoptotic marker	BCL2	Hs00608023_m1	BCL2	596
20.	Houskeeping control gene	actin beta	Hs01060665_g1	ACTB	60

Supplementary figure 2. **Real-time qPCR primer collection.**

**Positive Marker Cocktail**	CD105 PerCP-Cy5.5/CD73 APC/CD90 FITC
**Additional Positive Drop-In Marker**	CD44 PE
**Negative Marker Cocktail**	CD45/CD34/CD11b/CD19/HLA-DR PE
**Isotype Controls**	mIgG1, κ PerCP-Cy5.5/ mIgG1, κ APC / mIgG1, κ FITC (for positive cocktail)
	mIgG1 , κ/mIgG2a, κ PE (for negative cocktail)
	mIgG2b,κ PE (for CD44 drop in)

Supplementary figure 3. **Flow cytometry analysis antibody cocktails.**

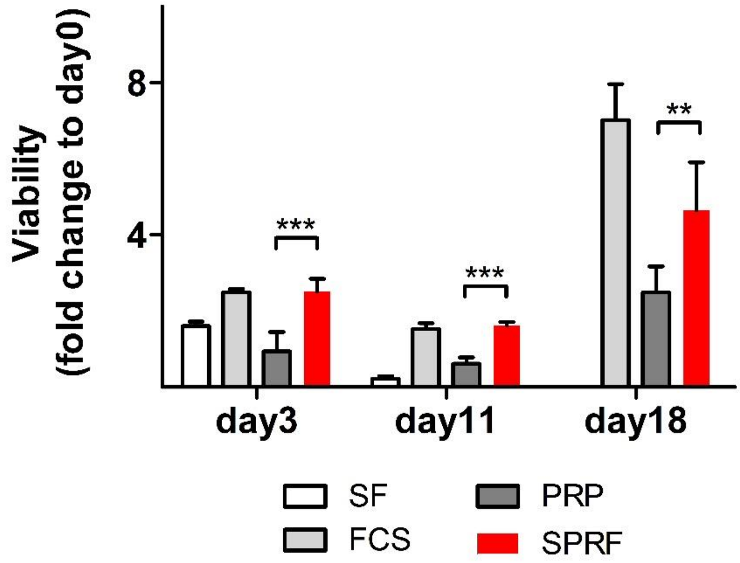

Supplementary figure 4. **Time-course effect of serum supplements on osteoblasts.** Subconfluent osteoblasts (LONZA, Walkersville, USA) were cultured in high glucose DMEM with pyruvate (Gibco, Paisley, Scotland) and with antibiotics (penicillin 200 U/mL; streptomycin 0.2 mg/mL, and amphotericin B 2.5 µg/mL) (Sigma-Aldrich, St. Louis, USA), and l-ascorbic acid (50 µg/mL; Sigma-Aldrich, St. Louis, USA). in the absence of supplement (□), 10 (v/v)% of FCS (□), 10 (v/v)% FCS + 1 ng/mL bFGF (■), 10 (v/v)% PRP (■) or 10 (v/v)% SPRF (■). XTT viability assay was performed on the third, 11th and 18th day. Results are presented as means of triplicate samples in three experiments ± SD. $p < 0.0001$ ***.

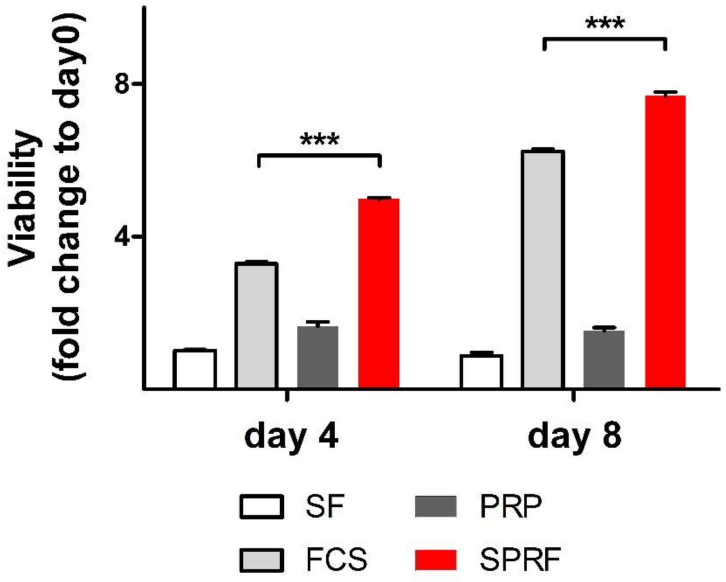

Supplementary figure 5. **Time-course effect of serum supplements on osteoarthritic chondrocytes.** Cells were cultured in DMEM/F12 (Gibco, Paisley, Scotland) with antibiotics (penicillin 200 U/mL; streptomycin 0.2 mg/mL, and amphotericin B 2.5 µg/mL) (Sigma-Aldrich, St. Louis, USA), and l-ascorbic acid (50 µg/mL; Sigma-Aldrich, St. Louis, USA), in the absence of supplement (□), 10 (v/v)% of FCS (□), 10 (v/v)% FCS + 1 ng/mL bFGF (■), 10 (v/v)% PRP (■) or 10 (v/v)% SPRF (■). XTT viability assay was performed on the 4th and 8th day. Results are presented as means of triplicate samples in three experiments ± SEM. p < 0,0001 ***.
